# Integrating ctDNA testing for *EGFR* analysis in advanced non-small cell lung cancer: strategies for clinical laboratories

**DOI:** 10.1515/almed-2025-0012

**Published:** 2025-05-01

**Authors:** Esther Fernández-Galán, Joan Anton Puig-Butillé

**Affiliations:** Department of Biochemistry and Molecular Genetics, 16493 Biomedical Diagnostic Center (CDB), Hospital Clinic de Barcelona, Barcelona, Spain; Fundació de Recerca Clínic Barcelona-Institut d’Investigacions Biomèdiques August Pi i Sunyer (IDIBAPS), University of Barcelona, Barcelona, Spain; Molecular Biology CORE, Biomedical Diagnostic Center (CDB), 16493 Hospital Clínic de Barcelona, Barcelona, Spain

**Keywords:** *EGFR*, liquid biopsy, ctDNA, lung cancer, serum tumor markers

## Abstract

Epidermal growth factor receptor gene (*EGFR*) molecular testing is essential for guiding targeted therapies in patients with advanced non-small cell lung cancer (NSCLC). Between 15 and 40 % of patients with NSCLC carry mutations in *EGFR* that are sensitive to tyrosine kinase inhibitors (TKIs). Due to the significant clinical benefits, identifying patients eligible for TKI therapy is crucial for optimizing treatment. While tumor tissue has been considered the gold standard for this testing, adequate material for *EGFR* molecular study cannot be obtained in up to 30 % of patients. In this context, circulating tumor DNA (ctDNA) analysis offers a guideline-recommended non-invasive method to detect *EGFR* mutations. Despite its promise, the widespread adoption of ctDNA analysis faces challenges for integration into clinical practice. This review provides a comprehensive synthesis of current knowledge on the clinical utility of *EGFR* molecular analysis in ctDNA, alongside its relationship with other circulating biomarkers widely implemented in clinical laboratories, such as serum tumor markers (STMs). It delves into the technical considerations, interpretation of results, and other challenges associated with ctDNA analysis, offering valuable insights into its integration into laboratory workflows.

## Introduction

Precision oncology has profoundly influenced the management of lung cancer (LC), which was the most frequently diagnosed cancer in 2022, responsible for almost 2.5 million new cases worldwide and the leading cause of cancer-related mortality [1]. Identification of molecular mechanisms involved in LC has led to the development and clinical implementation of targeted therapies, especially for non-small cell lung cancer (NSCLC).

The epidermal growth factor receptor (*EGFR*) gene represents the most prevalent and first targetable oncogenic gene in NSCLC. *EGFR* mutations occur in about 15–40 % of cases. EGFR tyrosine kinase inhibitors (EGFR-TKIs) have become the first-line systemic therapy for patients with EGFR-activating mutations as they lead to improvements in survival and quality of life [2]. Therefore, molecular testing is mandatory for advanced NSCLC to identify patients eligible for EGFR-TKI therapy [3].

However, the use of this testing in the clinical routine is often limited by the difficulty in obtaining adequate tumor tissue, which is considered the gold standard for molecular studies. In up to 30 % of LC patients, sufficient material for *EGFR* molecular analysis is unavailable [4]. Additionally, the invasive nature of tissue biopsies makes continuous monitoring impractical. In this context, “liquid biopsy” (LB) strategies have emerged as non-invasive or minimally invasive methods for analyzing tumor characteristics from biological fluids like blood [5]. These methods detect tumor-derived analytes, including circulating tumor DNA (ctDNA), circulating tumor cells, circulating free RNA, and extracellular vesicles.

This review will primarily focus on ctDNA analysis due to its extensive validation and broad acceptance in clinical settings. Analyzing *EGFR* mutations in ctDNA is an established method for identifying patients who would benefit from EGFR-TKIs. ctDNA testing is particularly valuable when tissue samples are unavailable or when fast turnaround time (TAT) is needed for urgent treatment decisions. However, the routine implementation of ctDNA analysis in clinical laboratories still faces significant challenges. Laboratory professionals play a crucial role in ensuring the reliability and optimal interpretation of these results, making them essential for successful integration into clinical practice.

This review aims to provide a comprehensive synthesis of current knowledge regarding the clinical utility of *EGFR* molecular analysis in ctDNA. From a clinical laboratory perspective, the review will explore technical aspects, clinical applications, benefits, and ongoing challenges associated with ctDNA analysis, while offering practical guidelines to effectively integrate ctDNA testing into routine practice.

## EGFR: structure, signaling and clinical implications

The EGFR is a 170-kDa transmembrane glycoprotein belonging to a family of four cell-surface receptors: (EGFR or HER1), HER2, HER3 and HER4. Early studies on the EGFR pathway were initiated with the discovery of epidermal growth factor (EGF) by Cohen and Levi-Montalcini in 1962 [6], [7]. Since then, biochemical and genetic studies have elucidated its role in regulating cellular growth, detailing the intricate cascade of intracellular signals following ligand-receptor interaction.

The oncoprotein EGFR is composed of three domains: an extracellular domain (rich in carbohydrates that facilitate ligand binding), a transmembrane domain (hydrophobic), and a cytoplasmic domain (with tyrosine kinase activity and phosphorylation sites). Ligands such as EGF and transforming growth factor α (TGFα) can bind to EGFR. Upon ligand binding, EGFR dimerizes, forming homodimers, and heterodimers with HER2 or other family members. This dimerization facilitates autophosphorylation, activating the downstream signaling [8]. The activation of this cascade induces cell proliferation and plays a significant role in cell survival and differentiation.

Therefore, EGFR overexpression or inappropriate activation can contribute to cancer development and progression. Increased EGFR signaling can result from *EGFR* gene amplification, protein overexpression, or specific activating mutations. EGFR is frequently overexpressed in various solid tumors, including lung, head and neck, breast, kidney, colon, ovarian, prostate, brain, and bladder cancers [9]. This overexpression has been associated with increased tumor aggressiveness and a reduced response to conventional therapies.

**Figure 1:**
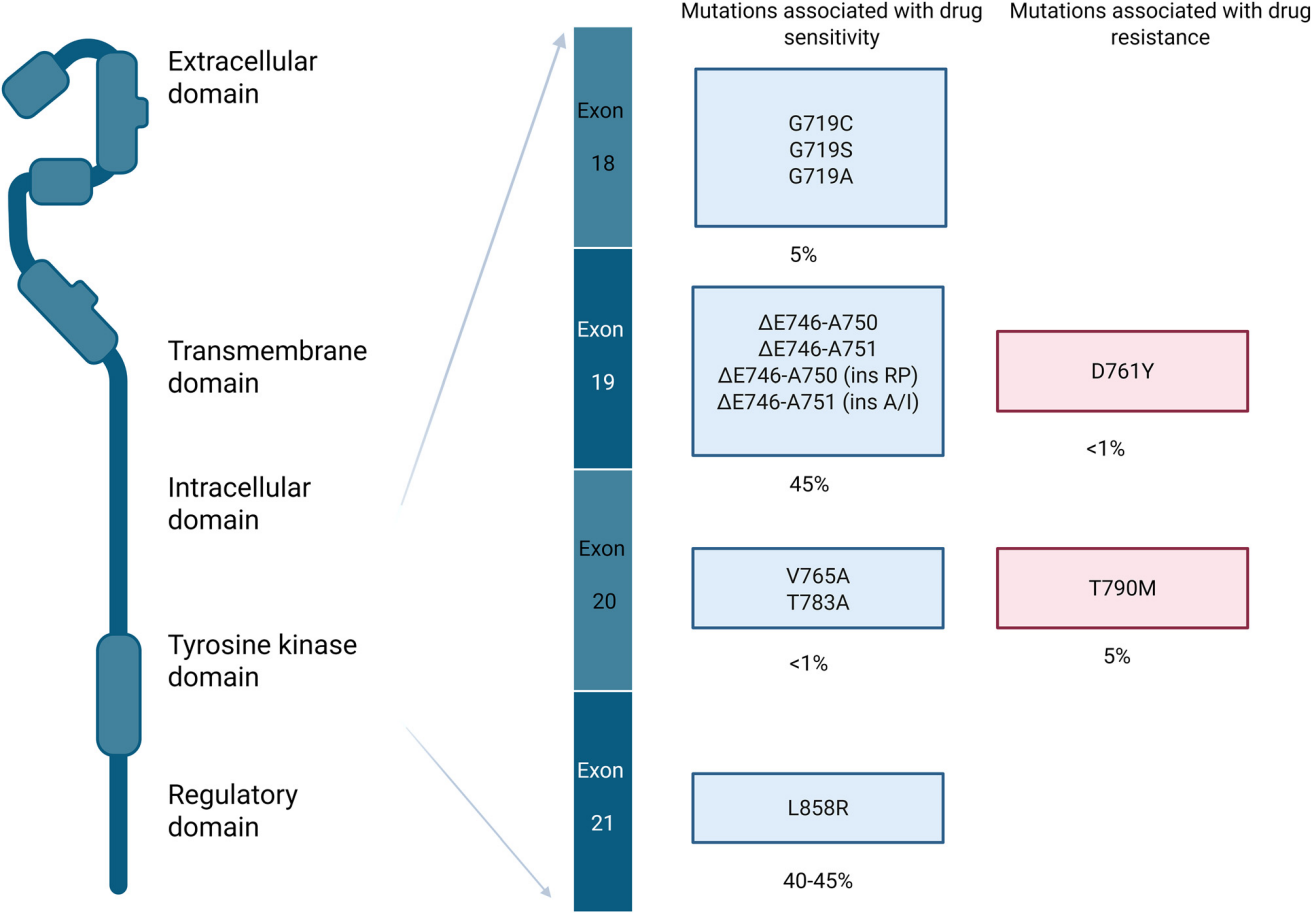
Clinical relevance of key *EGFR* mutations in non-small cell lung cancer. The Figure illustrates the clinical relevance of key *EGFR *mutations in non-small cell lung cancer (NSCLC). Mutations are grouped by their location within specific exons (18–21) and their association with drug sensitivity or resistance. Mutations in blue boxes are linked to increased drug sensitivity to EGFR tyrosine kinase inhibitors (TKIs), with their corresponding prevalence percentages noted. Mutations in red boxes are associated with drug resistance. Exon 19 deletions and the L858R mutation in exon 21 are the most prevalent drug-sensitive mutations, while T790M in exon 20 is the most common resistance mutation. The domains of the *EGFR* protein (extracellular, transmembrane, intracellular, tyrosine kinase, and regulatory) are also shown for structural context. Adapted from: Sharma SV, et al.: Epidermal growth factor receptor mutations in lung cancer, *Nat Rev Cancer* 7: 169–181, 2007 and Bai Y, et al.: Molecular genetics of solid tumors, in Henry’s Clinical Diagnosis and Management by Laboratory Methods, 24th ed, pp. 1579–1587, Elsevier, 2022. Figure created with Biorender.

In NSCLC, EGFR deregulation is prevalent, characterized by protein overexpression in 85 % of cases, gene mutations in 10–40 %, and gene amplification in approximately 10 % Protein overexpression (assessed by immunohistochemistry) or gene copy number (assessed by fluorescence *in situ* hybridization) lack consistent prognostic or predictive value. In contrast, the detection of *EGFR* mutations has proven clinical utility as biomarkers for predicting response to EGFR-TKIs [10]. These somatic mutations, which result in constitutive activation of EGFR kinase activity, are more frequent in women, patients with adenocarcinoma, Asian ethnicity, and those who have never smoked. The most common activating *EGFR* mutations in NSCLC are exon 19 deletions (>50 %) followed by the p.L858R point mutation in exon 21 (∼40 %), both of which are associated with a positive response to EGFR-TKI therapy. Mutations in exons 18 and 20 account for the remaining 10 % of *EGFR* mutations in NSCLC [11] (Figure 1).

The p.T790M point mutation represents the most common mechanism of acquired resistance, occurring in 50–60 % of patients treated with first- and second-generation EGFR-TKIs. In cases of atypical *EGFR* mutations, treatment response may vary compared to classical mutations. For instance, exon 20 insertions are typically associated with resistance to standard EGFR-TKIs, although the response can be heterogeneous [12], [13].

## Methodologies for molecular characterization of *EGFR* gene in circulating tumor DNA

Methodologies for detecting *EGFR* mutations in ctDNA can be categorized into two groups: targeted and non-targeted approaches (as detailed in Table 1). Targeted methods include real-time polymerase chain reaction-based techniques (RT-PCR), the amplification refractory mutation system (ARMS), and digital PCR (dPCR) methods such as droplet digital PCR (ddPCR) and BEAMing (beads, emulsions, amplification, and magnetics). These methods provide excellent specificity but are limited to detecting deletions and point mutations pre-defined in their design.

**Table 1:** List of methodologies used for *EGFR* molecular analysis in plasma (ctDNA).

Method	Mutations studied	LoD	CE-IVD	FDA	Ref.
**Targeted strategies**					

RT-PCR/ARMS					
EGFR mutation test v2 (Roche)	47 mutations: Exons 18, 19, 20, 21 + T790M	25–100 copies/mL	Yes	Yes	[14]
Idylla™ ctEGFR mutation assay (Biocartis)	49 mutations: Exons 18, 19, 20, 21 + T790M	<5 %	Yes	No	[15]
Therascreen EGFR plasma RGQ PCR kit (Qiagen)	29 mutations: Deletions Exon 19, L858R, T790M	0.81–17.5 %	Yes	No	[16]
ctDNA EGFR mutation detection kit (entroGen)	58 mutations: Exons 18, 19, 20, 21 + T790M + C797S	0.1–0.5 %	Yes	No	[17]
Easy EGFR (Diatech Pharmacogenetics)	T790M and C797S	<0.5 %	Yes	No	[18]
AmoyDx^®^ EGFR mutation detection test (Amoy diagnostics)	29 mutations: Exons 18, 19, 20, 21 + T790M	1 %	Yes	No	[19]
The SuperARMS EGFR mutation detection kit (Amoy diagnostics)	31 mutations: Exons 18, 19, 20, 21 + T790M	0.2–0.8 %	Yes	No	[20]
PANAMutyper-R-EGFR (Panagene)	29 mutations: Exons 18, 19, 20, 21 + T790M	0.1–0.5 %	Yes	No	[21]
dPCR and beaming					
ddPCR Custom assays (Bio-Rad)	L858R, T790M, C797S, deletions in exon 19	0.1–0.4 %	RUO	No	[22]
ddPCR EGFR Exon 19 deletions screening kit (Bio-Rad)	15 exon 19 deletions	<0.5 %	RUO	No	[23]
OncoBEAM EGFR kit v2 (Sysmex)	36 mutations, including T790M and C797S	0.1 %	RUO	No	[24]
Non-targeted Strategies^a^					
Oncomine precision assay (OPA) (Thermo Fisher Scientific)	DNA hotspots, CNV and intra-genic fusions	0.1–0.2 %	RUO	No	[25]
Oncomine™ lung cfDNA assay (Thermo Fisher Scientific)	T790M, C797S, L858R, exon 19 del	0.1 %	RUO	No	[26]
AVENIO ctDNA Targeted/Expanded/Surveillance kit v(Roche)	SNV, Indel, CNV	0.5–1%	RUO	No	[27]
TruSight Oncology 500 ctDNA v2 (Illumina)	Small variants, CNV, DNA fusions	0.2–0.5 %	RUO	No	[28]
Guardant360 CDx (Guardant Health, Inc)	SNV, InDels, CNV	0.2–1.8 %	Yes	Yes	[29]
FoundationOne liquid CDx (Foundation medicine, Roche)	Complete exonic coverage (substitutions, indels) and introns (7, 15, 24–27)	0.4–0.8 %	Yes	Yes	[30]

ARMS, amplification refractory mutation system; CE-IVD, *In Vitro* Diagnostic Medical Device (approved for use in the European Union); CNV, copy number variants; ddPCR, droplet digital PCR; dPCR, digital polymerase chain reaction; FDA, U.S. Food and Drug Administration (approved for clinical use in the United States); Indel, insertion/deletion mutation; LoD, limit of detection; RT-PCR, reverse transcription polymerase chain reaction; RUO, research use only; SNV, single nucleotide variant. Information derived from the manufacturer’s specifications. ^a^In broad coverage panels, information regarding mutation types is focused on the *EGFR* gene. The LoD ranges rely on cfDNA input and the type of variant analyzed.

Regarding analytical sensitivity, ARMS and RT-PCR are known for their fast and precise detection capabilities, with a limit of detection (LOD) ranging from 0.1 to 5 % in most commercial assays. These methods generate qualitative or semi-quantitative results. In contrast, dPCR offers enhanced sensitivity, achieving a LOD of 0.1–1 % by partitioning the sample into numerous discrete reactions. This approach minimizes background interference and improves the detection of low-abundance mutations. Unlike the previous methods, dPCR provides a direct quantitative result, enabling precise determination of variant allele frequencies (VAF).

Next-generation sequencing (NGS) offers a non-targeted approach that enables comprehensive genomic profiling, allowing for the detection of a wide range of mutations in *EGFR* and other relevant genes while providing quantitative results. NGS also allows the identification of novel mutations not detected by targeted assays. Despite historically facing challenges with lower sensitivity and specificity due to the inherent error rates in DNA polymerase and sequencing processes, advancements like deep sequencing, molecular barcoding, and error-correction algorithms have significantly improved their performance. These developments have led to unprecedented levels of sensitivity (LOD from 0.1 to 0.5 %).

In terms of turnaround time (TAT), the broader scope of NGS results in a longer TAT and requires complex computational analysis compared to targeted approaches such as RT-PCR or dPCR. While NGS methods have a TAT of 1–2 weeks, Idylla and other PCR methods have a TAT of approximately seven days [31], [32], [33]. In summary, while targeted approaches are highly effective for detecting specific mutations with low detection limits and fast TAT, NGS provides a more comprehensive overview of the mutational landscape.

According to the 2022 report from the European Molecular Quality Network (EMQN), targeted approaches are used by the majority of laboratories, with RT-PCR being the predominant method (51 %). Among these, the cobas^®^ EGFR Mutation Test v2 (Roche) was the most commonly employed assay, used by 27 % of all laboratories. This test was the first LB assay approved by the U.S. Food and Drug Administration (FDA) in 2016. Meanwhile, NGS strategies are also commonly adopted, with the Oncomine™ Lung cfDNA Assay (Thermo Fisher Scientific) being the most frequently used panel [34].

The rapid advancement of NGS technologies in recent years has significantly reduced sequencing costs while enhancing accuracy. Moreover, as the list of actionable gene alterations with FDA-approved therapies continues to grow (*EGFR*, *ALK*, *ROS1*, *BRAF*, *RET*, *MET*, *NTRK*, *KRAS*, *HER2*), there is a corresponding increase in the recommendation for broader NGS panels. These panels are not only more cost-effective than sequential single-gene testing in tissue samples but are also gaining prominence in plasma-based testing. The International Association for the Study of Lung Cancer (IASLC) and the European Society for Medical Oncology (ESMO) recommend adopting broad-based plasma ctDNA analysis by NGS, in advanced NSCLC [34], [35], [36]. In regions with a high prevalence of *EGFR* mutations, an initial targeted analysis remains pertinent as the first step in molecular testing. However, if a negative result is obtained by single-gene testing, it is advisable to proceed with serial testing for additional actionable biomarkers.

## Comparison of plasma ctDNA and tissue samples for *EGFR* molecular analysis

Tumor tissue has historically been considered the gold standard for *EGFR* molecular analysis. However, it has inherent limitations, including its invasive nature and challenges associated with obtaining biopsies from hard-to-reach locations or patients with complex clinical conditions. Additionally, tumor tissue samples may yield insufficient or degraded DNA, complicating the analysis. *EGFR* analysis in ctDNA obtained from plasma has shown high concordance with tissue samples, addressing some of these limitations. Plasma-based ctDNA analysis has several advantages: it is minimally invasive, demonstrates high concordance with tissue results, offers a rapid TAT and allows for repeated testing over time, providing a more comprehensive representation of tumor heterogeneity and clonal evolution. However, potential drawbacks include the risk of false negatives and higher costs when used alongside tissue testing [35].

When comparing plasma-based ctDNA testing with tumor tissue analysis, studies have shown a high level of concordance for *EGFR* mutations, as well as other guideline-recommended biomarkers [37], [38]. González de Aledo-Castillo et al. reported a concordance of 95.2 % for plasma cfDNA testing using the Cobas^®^ EGFR test in NSCLC patients at stages III and IV, compared with tissue analysis. In their prospective study, 39.9 % of patients lacked accessible tumor tissue for molecular characterization. In this scenario, plasma-based *EGFR* testing facilitated therapy initiation in 34.8 % of these patients, emphasizing the utility of ctDNA when tissue biopsy is not feasible [14].

Regarding the analytical performance, plasma-based ctDNA analysis generally demonstrates high specificity across platforms (>96 %). However, sensitivity varies considerably (58–100 %), depending on the platform and the mutations studied [35]. For instance, T790M mutations are typically detected at lower frequencies in plasma ctDNA compared to the primary sensitizing mutations (L858R or exon 19 deletions), leading to reduced sensitivity (41–90.5 %) [35]. A recent meta-analysis reported a pooled sensitivity of 68 % (95 % CI=60–75 %) and specificity of 98 % (95 % CI=95–99 %) [39].

Another significant advantage of plasma-based ctDNA analysis is the shorter TAT compared with tissue genotyping, which has a median TAT of 12 days (range 1–54 days) [40], [41]. Overall, employing a plasma-based strategy, significantly improves the availability of test results before the initial patient visit (85 vs. 9 %, p<0.0001), thus reducing time-to-treatment for patients with advanced NSCLC [42].

Plasma-based ctDNA analysis also offers the possibility of monitoring treatment response and disease progression longitudinally in patients receiving EGFR-TKIs [43]. Tissue re-biopsies can be risky due to the patient’s health status or tumor location; plasma analysis is a safer alternative to assess *EGFR* mutation status during treatment [44]. Additionally, ctDNA analysis can provide valuable insights into the mechanisms of resistance, such as MET amplification, revealing tumor heterogeneity and clonal evolution in osimertinib-resistant cases with *EGFR* mutations (AURA3 trial, NCT02151981) [45], [46]. Nevertheless, it is important to note that ctDNA analysis may not identify all resistance mechanisms, such as histologic transitions, which require morphological examination of tissue.

False negatives remain the weakness of ctDNA analysis; tissue biopsy remains necessary when LB tests yield negative results. Sensitivity may fluctuate based on both analytical and biological factors. Like other circulating markers, such as serum tumor markers (STMs), the biological characteristics of the tumor – such as stage, tumor burden, and vascularization – can influence the amount of ctDNA released into circulation, which impacts sensitivity. Tumors with low ctDNA shedding are observed in 15–32 % of NSCLC patients [47], and sensitivity is further reduced in early-stage NSCLC, dropping below 30 % [48]. False negative results can also arise from technique’s limitations, underscoring the increasing focus on high-sensitive methods like NGS. Some studies have explored alternative matrices, such as bronchial washing fluid, which has shown significantly higher sensitivity for detecting *EGFR* mutations than plasma [49], [50]. False positives are uncommon but can occur, particularly when using broad-spectrum methods like NGS, which analyze multiple genes. These false positives can arise from germline variants or non-tumor sources such as clonal hematopoiesis of indeterminate potential (CHIP). While CHIP can influence mutations in genes like *TP53* and *KRAS*, it does not notably impact the analysis of *EGFR* mutations [51].

In conclusion, *EGFR* analysis in plasmatic ctDNA offers significant advantages in terms of non-invasiveness, accessibility, and monitoring capability, but it also has limitations. Plasma ctDNA analysis does not replace tissue genotyping and should be considered a complementary tool in the therapeutic decision-making for advanced NSCLC.

## Preanalytical and postanalytical factors

The pre-analytical phase plays a critical role in influencing cfDNA results. Adhering to established guidelines for sample collection, handling, transport, and cfDNA extraction is essential to achieve optimal standardization and reliability [52]. The main challenges affecting the quality of cfDNA analysis include: (1) the heterogeneous content of blood, which complicates cfDNA isolation, (2) enzymatic degradation and clotting, (3) the instability of naked DNA in biological environments, (4) contamination of cfDNA by genomic DNA (gDNA), and (5) the difficulty in detecting the small, specific fraction of ctDNA within the cfDNA pool.

Regarding cfDNA isolation, plasma is the preferred specimen over serum to avoid contamination with gDNA from disrupted leukocytes during clotting [53]. Blood should be collected in tubes with cell stabilizer (e.g. Streck^®^) or EDTA, which is the recommended anticoagulant. When EDTA is used, plasma isolation should be performed within 4 h [53]. When samples need to be transported to different centers, it is preferable to use commercial cell stabilizer tubes, as they prevent blood cell lysis and allow processing times to be extended for several days at room temperature. A two-step plasma centrifugation process is recommended: first, low-speed centrifugation to concentrate blood cells, followed by high-speed centrifugation to remove cell debris. For the extraction method, ready-to-use automated kits are preferred for routine applications [52]. It is also advised to perform quality control on the cfDNA extract to assess both cfDNA quantity and fragmentation levels [54].

In the post-analytical phase, the interpretation and reporting of *EGFR* variants in ctDNA should be consistent with standard criteria for somatic variant interpretation [55], [56]. It is also crucial to consider the unique characteristics of ctDNA and adhere to specific guidelines for ctDNA analysis [37], [57], ensuring accurate detection and reporting of *EGFR* mutations relevant to NSCLC.

Molecular reports for cfDNA should be clear and concise, emphasizing clinically significant information. Genetic alterations should be described using standardized nomenclature as per Human Genome Variation Society (HGVS) guidelines (http://varnomen.hgvs.org). Additionally, reports should include simplified, colloquial terms to enhance clarity and understanding. For instance, an *EGFR* gene alteration should be listed as “c.2369C>T (p.Thr790Met)” and can optionally be described as the “T790M mutation”. Reports should provide essential details to guide clinical decision-making, emphasizing the clinical significance of identified variants concerning FDA/EMA-approved therapies. Key methodological information should be included at the end of the report: the alterations tested, limitations, allelic frequencies, coverage, and other pertinent technical data. The language used in reporting should highlight the possibility of discrepancies with tumor testing, particularly in situations where a variant is not identified in plasma ctDNA [37]. Considering the potential risk of false negatives, *EGFR* results in ctDNA should not be reported merely as negative. Instead, they should be described as “non-informative” or “not detected”. This terminology reflects the possibility that an *EGFR* mutation may be present in the tissue but not detectable in the plasma due to biological factors or technical limitations.

Finally, Molecular Tumor Boards are crucial for interpreting complex cases, requiring the collaboration of multidisciplinary experts. These boards ensure a thorough evaluation of molecular findings, with laboratory professionals playing a crucial role in ensuring the correct interpretation of molecular alterations, especially when addressing variants of uncertain significance or conflicting results.

## Clinical utility and guideline recommendations across diverse clinical scenarios

National and international guidelines recommend ctDNA genotyping as a surrogate for tissue genotyping in the molecular analysis of *EGFR* and other relevant biomarkers in advanced NSCLC [58], [59]. This approach is advised when tissue samples are unavailable, both for initial diagnosis and for monitoring resistance. Decision algorithms, provided by organizations such as IASLC, help guide the selection of plasma- or tissue-based testing strategies. IASLC recommends a “plasma-first” strategy when a tissue sample is unavailable, a “complementary approach” when tissue is available but is scant or of uncertain adequacy for genotyping, and a “sequential approach” when tissue genotyping is incomplete [35].

By contrast, tissue-based testing remains the preferred method for early stages NSCLC (stages I–III), which accounts for 25–30 % of patients. Osimertinib, a third-generation EGFR-TKI, was approved by the FDA in 2020 to reduce the recurrence rate post-surgery in patients with stage IB to IIIA NSCLC with *EGFR* activating mutations (*EGFR*+), based on the ADAURA trial, which demonstrated significantly improved disease-free survival (DFS) [60]. In the neoadjuvant setting, anti-EGFR therapies may reduce tumor size and improve resection rates, though, ongoing trials like NeoADAURA are critical to further refining treatment strategies in this context.

In early-stage NSCLC, ctDNA is not routinely recommended, and its clinical utility remains under investigation. Its potential lies in its value as a prognostic biomarker for assessing the risk of recurrence. Many patients experience recurrence following surgery, and post-surgical cfDNA monitoring, offers an opportunity for earlier detection, enabling prompt intervention or closer follow-up before radiological signs of recurrence appear. In NSCLC-*EGFR*+ patients, the presence or absence of ctDNA before and after surgery is a strong prognostic indicator, with ctDNA negativity or clearance being associated with better DFS. Notably, most patients with undetectable minimal residual disease through longitudinal ctDNA monitoring did not experience recurrence [61].

In the screening context, there is a critical need for non-invasive screening methods to improve early detection of LC in high-risk and general populations. Research into circulating biomarkers, including proteins, autoantibodies, gene expression profiles, and microRNAs, shows promise for early detection and distinguishing cancer from non-cancerous nodules [62]. Currently, none of these approaches are approved for clinical use. Blood tests utilizing LB techniques can identify various surgically resectable cancers by detecting cfDNA mutations in key oncogenes, such as *EGFR*, and analyzing circulating proteins. However, their sensitivity for detecting these alterations in early-stage lung cancer (LC) remains relatively low [63].

Combining different biomarkers and techniques with clinical features offers the most promising strategy to increase detection sensitivity and specificity. A recent study demonstrated that integrating cfDNA fragmentation analysis with clinical risk factors and STM concentration (CEA) significantly enhanced LC detection across various stages and subtypes, including 91 % of stage I/II and 96 % of stage III/IV, at 80 % specificity [64].

## Correlation between *EGFR s*tatus and serum tumor markers

Tumoral circulome represents the analysis of genetic biomarkers (cfDNA) in addition to other tumor-derived biomarkers, such as proteins and other molecules released into circulation. This novel multi-omic approach is gaining interest in the precision oncology field [65]. STMs are glycoproteins secreted into the bloodstream from the tumor cells or tumor microenvironment. STMs are routinely measured in clinical laboratories for the management of epithelial neoplasms; however, their clinical utility in LC remains controversial. Prospective studies have shown that combining six STMs (CEA, CYFRA 21.1, CA 15.3, SCC, NSE, and ProGRP) with clinical parameters represents the most accurate approach for detecting LC in symptomatic patients [66]. Additionally, recent findings indicate that integrating STM with ctDNA analysis is a promising strategy for early diagnosis of LC [63], [67]. The combination of STMs and ctDNA has predictive value, with elevated levels being linked to poorer prognosis and shorter progression-free survival following EGFR-TKI treatment [68]. However, there is currently insufficient evidence to include none of these blood biomarkers in clinical guidelines for LC diagnosis.

Regarding molecular features, several STMs have been linked to *EGFR* mutation status or other molecular alterations, as summarized in Table 2. Most studies have found an association between elevated levels of CEA and CA 15-3, and/or decreased levels of CYFRA 21-1 and SCC, with *EGFR* mutations [69], [70], [71], [72], [73], [74], [75]. This aligns with evidence suggesting that specific markers predict certain histology: CEA and CA 15-3 for adenocarcinoma, where *EGFR* mutations are more prevalent, and CYFRA 21-1 and SCC for squamous cell carcinoma [66]. By contrast, other studies found no significant differences between *EGFR* status and STM [76], [77], [78]; particularly in early-stage patients where expression in circulation and, therefore, the sensitivity is low [70]. During follow-up of EGFR-TKI therapy, dynamic STMs may predict the effectiveness of treatment [79], including the presence of secondary T790M mutation.

**Table 2:** Summary of studies evaluating the association between serum tumor markers and *EGFR* molecular status.

Title	Cohort	STMs	Main conclusions	Year	Ref
Distinction of *ALK* fusion gene and *EGFR* mutation-positive lung cancer with tumor markers	306 LC Stage III/IV 43.8 % *ALK*+ 56.2 % *EGFR*+	CEA CYFRA 21-1	Higher proportion of *ALK*+ patients were CYFRA21-1 positive Higher CYFRA 21-1: CEA ratios were observed in *ALK*+ patients compared to *EGFR*+ patients.	2024	[69]
The predictive value of serum tumor markers for *EGFR* mutation in non-small cell lung cancer patients with non-stage IA	6711 NSCLC Stage IA and non-stage IA 57.9 % *EGFR*+	CEA CYFRA 21-1 SCC	Significant associations with *EGFR* mutations in non-stage IA. No STM predictors in stage IA. Combination of STMs with clinical factors effectively predicts EGFR mutations.	2024	[70]
Dynamic monitoring serum tumor markers to predict molecular features of *EGFR-* mutated LC during targeted therapy	303 LC Stages III–IV 43 % *EGFR*+	CEA CYFRA 21-1 NSE CA125 CA153 SCC ctDNA	*EGFR* mutations: Significantly associated with female gender, abnormal CEA and CA153 levels, and normal SCC level. T790M mutation: More common in patients with abnormal CEA levels. Baseline STM levels and variations: May suggest secondary T790M mutation	2022	[71]
Establishment and evaluation of *EGFR* mutation prediction model based on Tumor markers and CT features in NSCLC	148 NSCLC Stages I–IV 51 % *EGFR*+	CEA CYFRA 21-1 NSE CA 125 CA 19.9	Predictive factors for *EGFR* mutation: Non-smoking status, high CEA and low CYFRA21-1 CYFRA21-1 levels: Higher in the wild-type group, CA 19.9 levels: Higher in the *EGFR* mutation group.	2022	[72]
Value of serum tumor markers for predicting *EGFR* mutations and positive *ALK* expression in 1089 Chinese non-small-cell lung cancer patients: A retrospective analysis	1089 NSCLC *EGFR* status 50.1 % *EGFR*+ (1088 evaluated)	CEA CA 125 SCC CYFRA 21-1 FERR	*EGFR* mutations associated with ADC, never-smoker status, and negative CA 125, SCC, FERR, CYFRA 21-1. Multivariate analysis demonstrated that ADC, never-smoker status, and negative CA 125 and SCC results were predictors of *EGFR* mutations	2020	[73]
Value of serum tumor markers for predicting *EGFR* mutations in non-small cell lung cancer patients	143 NSCLC 44.06 % *EGFR+*	CEA CYFRA 21-1 NSE SCC ProGRP	STMs are associated with mutant *EGFR* status and could be integrated with other clinical factors to facilitate the classification of *EGFR* mutation status among NSCLC patients. Univariate logistic regression analysis showed that gender, smoking status, histological type, SCC, and proGRP levels were significantly correlated with *EGFR* mutations.	2020	[80]
Combining PET/CT with serum tumor markers to improve the evaluation of histological type of suspicious lung cancers	201 LC Stages I–IV 50 % *EGFR*+ (16 evaluated)	CEA CYFRA 21-1 NSE SCC	No significant difference between different *EGFR* mutation statuses in SUVmax, CEA, CYFRA 21-1, SCC-Ag or NSE	2017	[76]
Predictive and prognostic value of CYFRA 21-1 for advanced non-small cell lung cancer treated with EGFR-TKIs	95 NSCLC Stages IIB-IV 57 % *EGFR*+	CEA CYFRA 21-1	Serum CYFRA 21-1 level may be a predictive factor for patients with NSCLC treated with EGFR-TKIs, regardless of *EGFR* mutation status. Elevated serum CYFRA 21-1 was associated with shorter PFS and OS of patients with NSCLC treated with EGFR-TKI.	2017	[79]
Correlation between EGFR gene mutation, cytologic tumor markers, 18F-FDG uptake in non-small cell lung cancer	61 NSCLC Stages I-IV 49.1 % *EGFR*+	Serum and cytologic CEA CYFRA 21-1 SCC	No significant difference in STM levels between wild-type and mutant *EGFR*. c-CYFRA levels: Significantly higher in patients with *EGFR* mutations compared to those with wild-type *EGFR*.	2016	[77]
Predictive and prognostic value of preoperative serum tumor markers is *EGFR* mutation-specific in resectable non-small-cell lung cancer	1016 NSCLC I–IIIA 25 % *EGFR*+ (979 evaluated)	CEA CYFRA 21-1 SCC NSE	There is no difference in CEA or CYFRA21-1 levels between *EGFR*-positive and wild-type adenocarcinoma patients. CYFRA21-1 serves as a predictive and prognostic marker in resectable adenocarcinoma patients with *EGFR* mutations, particularly in the *EGFR* del19 or L858R groups. CEA is an independent predictive and prognostic factor only for *EGFR* wild-type adenocarcinoma and *EGFR* L858R adenocarcinoma patients.	2015	[78]
Monitoring of carcinoembryonic antigen levels is predictive of EGFR mutations and efficacy of EGFR-TKI in patients with lung adenocarcinoma	70 ADC Stage IIA–IV 62.9 % *EGFR*+	CEA	High-level CEA is independently associated with *EGFR* gene mutation The variation types of CEA level could help us to predict the efficacy of EGFR-TKI in patients harboring *EGFR* mutation within only one month of TKI therapy	2014	[74]
Correlation between *EGFR* mutations and serum tumor markers in lung adenocarcinoma patients	70 ADC Stages I–IV 38.6 % *EGFR*+	CEA CA 242	Serum CEA and CA242 levels are associated with the presence of *EGFR* mutations.	2013	[75]

For studies where the stage is not provided, this information is not specified in the cohort section. ADC, adenocarcinoma; ALK, anaplastic lymphoma kinase; CEA, carcinoembryonic antigen; CA125, carbohydrate antigen 125; CA153, carbohydrate antigen 153; CA 242, carbohydrate antigen 242; CYFRA 21-1, cytokeratin-19 fragments; c-CYFRA 21-1, cytologic CYFRA 21.1; ctDNA, circulating tumor DNA; EGFR, epidermal growth factor receptor; FERR, ferritin; LC, lung cancer; NSE, neuron-specific enolase; OS, overall survival; PFS, progression free survival; SCC, squamous cell carcinoma antigen; STM, serum tumor markers; TKI, tyrosine kinase inhibitor.

All this evidence suggests that STMs could be integrated with other clinical factors to predict *EGFR* status and combined ctDNA + STMs monitoring can offer insights into therapy response, enhancing personalized treatment decisions. However, there is considerable variability among studies highlighting the need for ongoing research into their clinical utility.

A promising strategy can be driven by recent advances in artificial intelligence and machine learning, enabling the efficient combination of markers into multi-marker models, integrating clinical and imaging data. This strategy facilitates the analysis of large volumes of clinical and imaging data to identify patterns that can predict the presence and progression of cancer [81], [82]. These advancements pave the way for the development of clinical decision support tools and open up new opportunities for more effective, personalized cancer treatments.

## Conclusions

This review provides practical guidelines for integrating ctDNA analysis into real-world clinical settings. *EGFR* mutations are critical therapeutic targets in advanced NSCLC, and the limited availability of tissue samples highlights the need for non-invasive approaches, such as ctDNA analysis in plasma. Current guidelines recommend ctDNA genotyping as a surrogate for tissue genotyping when tissue samples are unavailable, with decision algorithms guiding the choice between plasma- or tissue-based testing strategies.

Clinical laboratories play a crucial role in ensuring reliable molecular analysis of *EGFR* in ctDNA, addressing both pre-analytical and post-analytical phases. While targeted methods remain the most common approach in clinical practice due to their robustness and rapid TAT, NGS based methods are becoming increasingly important for more comprehensive mutational profiling. This shift is driven by the evolving landscape of targeted therapies and the growing need for comprehensive testing to identify resistance mechanisms.

Beyond its established role in advanced NSCLC, ctDNA analysis is being explored for earlier stages of the disease. The potential use of EGFR-TKI therapies in both neoadjuvant and adjuvant settings highlights the need for non-invasive *EGFR* testing in early-stage NSCLC to guide targeted treatment. While ctDNA analysis has shown prognostic utility post-surgery, its application in early stages and LC screening is currently limited by sensitivity issues. Tumoral circulome analysis, which integrates molecular alterations, cfDNA fragmentation, proteins and other biomolecules released by tumor cells into the circulation, is emerging as a promising non-invasive method. This approach could significantly enhance early detection sensitivity and precision, as well as improve personalized treatment strategies.
